# Differences in the fungal communities nursed by two genetic groups of the alpine cushion plant, *Silene acaulis*

**DOI:** 10.1002/ece3.4606

**Published:** 2018-11-21

**Authors:** Julien Roy, Jean‐Marc Bonneville, Patrick Saccone, Sébastian Ibanez, Cécile H. Albert, Marti Boleda, Maya Gueguen, Marc Ohlmann, Delphine Rioux, Jean‐Christophe Clément, Sébastien Lavergne, Roberto A. Geremia

**Affiliations:** ^1^ Laboratoire d’Ecologie Alpine (LECA) University Grenoble Alpes University Savoie Mont Blanc CNRS, LECA Grenoble France; ^2^ Present address: Institut für Biologie, Ökologie der Pflanzen Freie Universität Berlin Germany; ^3^ Present address: Centre for Polar Ecology University of South Bohemia Ceske Budejovice Czech Republic; ^4^ Present address: Aix Marseille Univ, Univ Avignon, CNRS, IMBE Marseille France; ^5^ Present address: CARRTEL, INRA – Université Savoie Mont Blanc Thonon‐les‐Bains France

**Keywords:** community genetics, fungal community, gene‐for‐gene interactions, nurse effect, soil ecosystem engineering

## Abstract

Foundation plants shape the composition of local biotic communities and abiotic environments, but the impact of a plant's intraspecific variations on these processes is poorly understood. We examined these links in the alpine cushion moss campion (*Silene acaulis*) on two neighboring mountain ranges in the French Alps. Genotyping of cushion plants revealed two genetic clusters matching known subspecies. The *exscapa* subspecies was found on both limestone and granite, while the *longiscapa* one was only found on limestone. Even on similar limestone bedrock, cushion soils from the two *S. acaulis* subspecies deeply differed in their impact on soil abiotic conditions. They further strikingly differed from each other and from the surrounding bare soils in fungal community composition. Plant genotype variations accounted for a large part of the fungal composition variability in cushion soils, even when considering geography or soil chemistry, and particularly for the dominant molecular operational taxonomic units (MOTUs). Both saprophytic and biotrophic fungal taxa were related to the MOTUs recurrently associated with a single plant genetic cluster. Moreover, the putative phytopathogens were abundant, and within the same genus (*Cladosporium*) or species (*Pyrenopeziza brassicae*), MOTUs showing specificity for each plant subspecies were found. Our study highlights the combined influences of bedrock and plant genotype on fungal recruitment into cushion soils and suggests the coexistence of two mechanisms, an indirect selection resulting from the colonization of an engineered soil by free‐living saprobes and a direct selection resulting from direct plant–fungi interactions.

## 1 INTRODUCTION

Terrestrial plants interact with aboveground and belowground fungal communities (Buée et al., 2009; Jumpponen & Jones, 2009) whose assemblage is influenced both by abiotic factors and by the plant community. Through positive or negative plant–soil feedbacks, fungi, in turn, shape plant community composition (Bever, Platt, & Morton, 2012), playing a major role in their evolution (Brundrett, 2002) and in ecosystem functioning (Berendsen, Pieterse, & Bakker, 2012). Vascular plants provide dead or living organic matter to most soil fungi; thus, they interact directly with biotrophic fungi but also modify their edaphic environment (De Deyn, Cornelissen, & Bardgett, 2008), hence, impacting the saprotrophic community (Millard & Singh, 2010).

Distance can limit fungal distributions since they present biogeographic patterns at the continental (Prober et al., 2015; Talbot et al., 2014; Tedersoo et al., 2014) and regional scales (Geremia, Pușcaș, Zinger, Bonneville, & Choler, 2016; Pellissier et al., 2014). Locally, the available pool of fungi then undergoes selection by soil‐ or plant‐related parameters, including aboveground plant species composition. Fungal community composition is correlated with soil moisture (Hawkes et al., 2011) or pH (Rousk et al., 2010), but also with plant legacies, such as soil C/N ratio (Prober et al., 2015) or soil organic matter content (Zinger et al., 2011). The soil fungal communities are further linked to the present plant communities, and fungal beta diversity (compositional dissimilarity between sites, see Anderson et al., 2011) is correlated with plant beta diversity (Geremia et al., 2016; Pellissier et al., 2014; Prober et al., 2015; Tedersoo et al., 2014). Also, different plant species give rise to diverging fungal communities from the same starting soil in controlled experiments, likely due to differences in rhizodeposition (Mouhamadou et al., 2013). Plant intraspecific genetic variation can also lead to divergent composition of dependent communities (Bangert & Whitham, 2007; Bangert et al., 2006). For endophytic fungi, this may be mediated directly by the differential production of defense compounds as shown in maize (Saunders & Kohn, 2009) or in *Populus* (Bailey et al., 2005; Lamit et al., 2014). However, little is known about the influence of plant genetic variation on fungal community composition in soils, which is challenging to disentangle from confounding factors in natural environments. Here, we report on a field survey providing insights into this influence.

Alpine cushion plants offer an interesting system to study fungal community assembly and to assess the role of intraspecific plant genetic variation. These foundation plants locally modify their physicochemical environment (Badano, Jones, Cavieres, & Wright, 2006) and the soil biotic community (Roy et al., 2013), both defining ecological engineering effects (see Jones, Lawton, & Shachak, 1994). Indeed, once a plant is established in cracks on bare mineral soil, the accumulating litter material below the dense green tissues allows the constitution of a de novo organic soil. Through the buffering of microclimatic conditions (Molenda, Reid, & Lortie, 2012) and the improvement of soil organic matter and nutrients status (Roy et al., 2013), cushion plants act as nurse species for microbiota (Roy et al., 2013), soil arthropods (Maillet, Lemaitre, Chikhi, Lavenier, & Peterlongo, 2012; Molenda et al., 2012), and other plants (Butterfield et al., 2013). To our knowledge, however, the extent of the abiotic and biotic soil changes induced by various cushion plant species or subspecies has not been explicitly studied.

**Figure 1:**
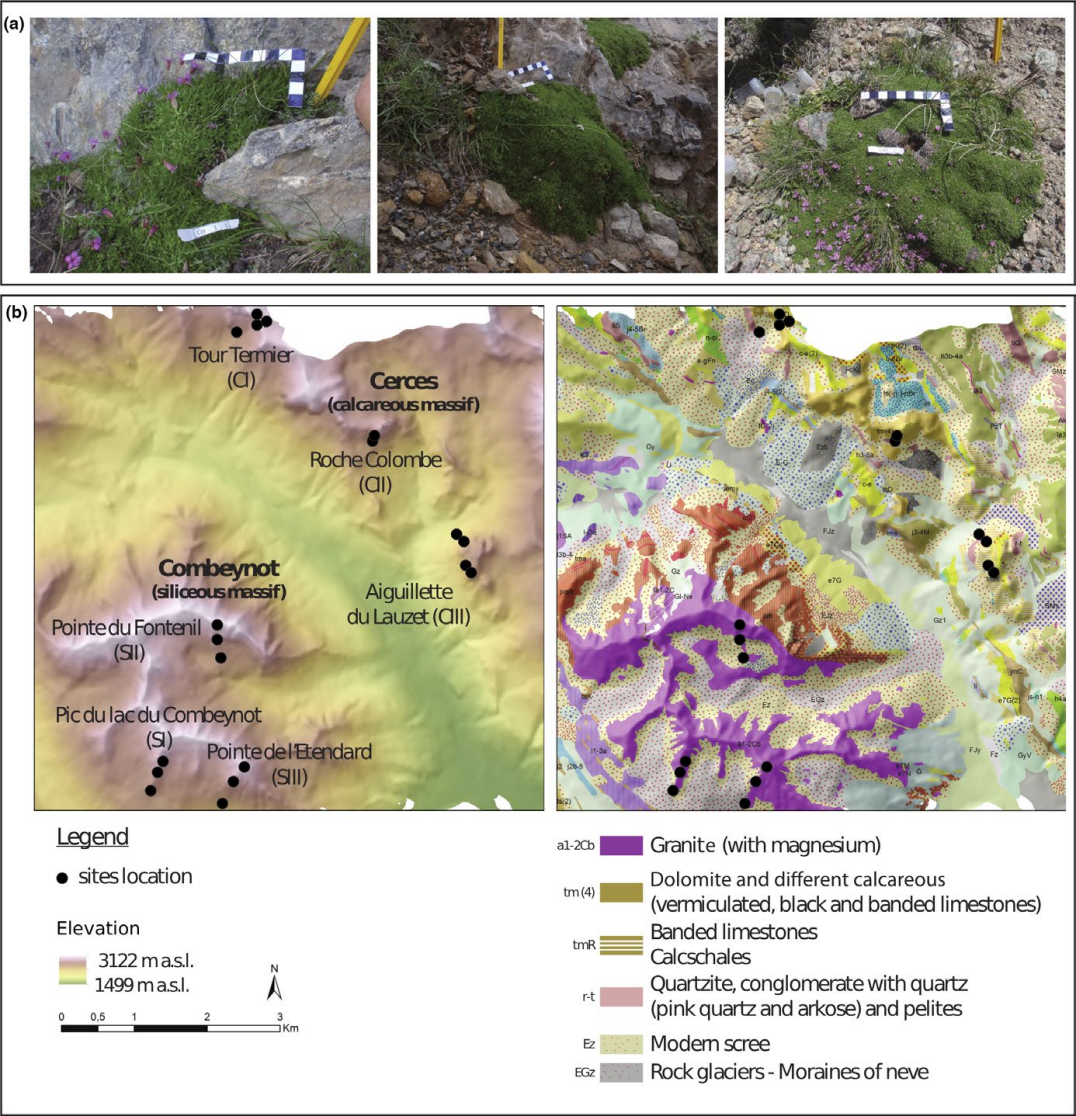
Morphology variation in *Silene acaulis* and sampling design. (a) *S. acaulis* cushions vary in shape from loose (left) to intermediate (middle) to dense cushions (right). The scale bar unit is 1 cm. (b) Topographical and geological maps showing the sampling sites along the six elevation gradients. Only bedrock types where a plant population was sampled are displayed in the legend

*Silene acaulis *(L.) Jacq is a common circumboreal and alpine cushion plant with slow growth and cushions that can often be older than 300 years (Morris & Doak, 1998). Its xenogamic reproduction makes that two distinct plant individuals always represent distinct genotypes. Although the evolutionary history of *Silene acaulis* remains to be fully unraveled, two main subspecies have been proposed in the Alps, *S. acaulis exscapa *and *S. acaulis longiscapa*, exhibiting dense‐ (shorter internodes) and loose‐cushion morphologies, respectively (Aeschimann, Lauber, Moser, & Theurillat, 2004; Bock, 1983). They occur on adjacent territories, probably with genetic barriers*, longiscapa* being restricted to calcareous bedrock while *exscapa* is mainly, but not only, found on siliceous bedrock (Sébastien Ibanez, S. I., unpublished obs.). Subspecies determination can, however, be difficult since intermediate cushion architectures may sometimes be found (Figure 1a).

We previously reported that the presence of a cushion affects soil chemical parameters on two bedrocks and that this shift in abiotic properties comes along with a shift in the fungal community; in particular, the fungal turnover from bare soil to cushion soil increased with environmental stress (high elevation and very low pH; Roy et al., 2013). In the present study, we first established that the two plant subspecies corresponded to distinct genotypic clusters, which then allowed us to assess the correlation between plant genetic distances and fungal beta diversity. To disentangle this genetic effect from those of the local environment (geology, elevation), and also to measure abiotic soil engineering by the plant, we sampled soils both beneath cushions and in neighboring bare soils. We finally examined the fungal functional guilds across the sampling design to address the distribution, filtering, and recruitment mechanisms.

## 2 MATERIALS AND METHODS

### 2.1 Plant and soil sampling

**Figure 2:**
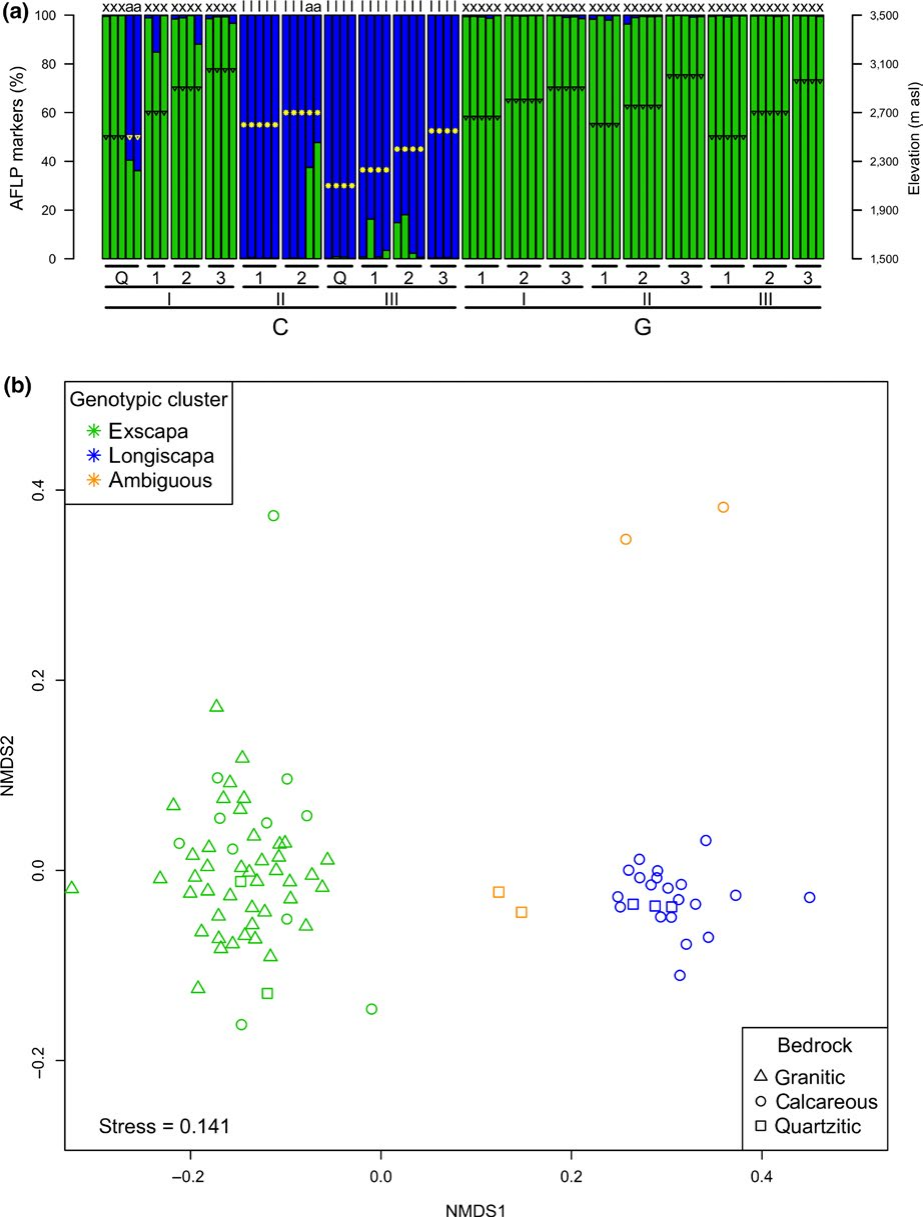
Plant genetic groups and their geographic distribution. (a) Proportion of AFLP alleles on plant individuals along the sampling gradients. Colors and letters refer to genotypic clusters: “*x,*” “*l,”* and “*a”* for *exscapa, longiscapa, *and ambiguous plants, respectively, as assigned by STRUCTURE (K = 2). The gradient of origin, elevation, and morphology of the sampled plants (triangles for dense and circles for loose cushions) are indicated; letters “C” (calcareous) and “G” (granitic) indicate the bedrock type (except “Q”, quartzitic). (b) NMDS plot of the Jaccard distances between AFLP fingerprints of cushion plants. Colors denote genotypic clusters, and shapes denote the bedrock of origin

The sampling area was located in the French Hautes‐Alpes along replicated ecotones from alpine to subnival environments and encompassed two neighboring mountain massifs: one mostly calcareous (Cerces) and one siliceous (Combeynot; see Roy et al., 2013 for details). In September 2009, at 19 sites located across six elevation gradients (Figures 1b and 2a, Supporting Information Table S1), with each site defining a plant population, we sampled cores of organic soil (5 cm in diameter x 5 cm deep) beneath five *S. acaulis* cushion plants, and five soil cores on neighboring bare ground. Both types of soil cores were split into two aliquots. One aliquot was frozen for physicochemical characterization. A second one was kept in silica gel until DNA extraction for fungal sequencing. Sites were revisited in July 2010 for plant leaf sampling.

*Silene acaulis* is a gynodioecious species, but plant gender was not recorded since some individuals were not flowering. Vegetative traits are not distinguishable between plant genders, and pollinators visit both plant genders, thus disseminating the same fungal spores; therefore, we assume that gender will be only a weak source of fungal community variation compared to plant genotype or environment. Plant material was kept in silica gel until DNA extraction for genotyping. Altogether, the sampling consisted of 95 leaf samples and 95 pairs of cushion and bare soil samples. Whereas the Combeynot massif is entirely siliceous (i.e., granite and gneiss), the Cerces massif is mostly composed of carbonated sedimentary rocks (hereafter referred to as limestone), but also contains quartzite (Figure 1b). Even though quartzite and granite are both siliceous bedrocks, the pH of quartzite samples was atypical (Supporting Information Table S1), and the two populations on quartzite were withdrawn from most analyses, leaving nine populations on granite and eight on limestone.

### 2.2 Soil physicochemical analyses

Soil chemical parameters were quantified following the methods described in Clément et al. (2012). Briefly, soil samples were sieved at 2 mm and used to estimate soil pH in water, gravimetric soil water content (Robertson, Coleman, Bledsoe, & Sollins, 1999), and to extract NO_3_^‐^ and NH4^+^ for analysis on a Flow Solution IV colorimetric chain (OI AnalyticalCorp, College Station, TX, USA). Total soil nitrogen and carbon contents were measured on air‐dried and ground soil samples with a FlashEA 1112 CN elemental analyzer (Thermo Fisher Scientific, Waltham, MA, USA). A principal component analysis (PCA; Legendre & Legendre, 2012) was performed to summarize the abiotic variations associated with the presence and genotype of cushion plants, using centered and reduced soil chemical data in *ade4* (Dray & Dufour, 2007).

### 2.3 Plant genotyping

Plants genotypes were fingerprinted using AFLP (Meudt & Clarke, 2007) by the procedure of Vos et al. (1995) with minor modifications. Total DNA was extracted from leaf tissues using the DNeasy 96 Plant Kit (Qiagen, Hilden, Germany) following the manufacturer's instructions, and plant DNA was digested for 2 hr at 37°C in a 20‐µl mix using 2 U of Mse1 and 5 U of Eco*R*I (New England Biolabs, Ipswich, MA, USA). Double‐stranded adaptors were ligated to the digested DNA in a 40 µl volume for 2 hr at 37°C using 1 U of T4 DNA Ligase (Roche). Products were then diluted to 1:10, and a preselective PCR (2 min at 72°C, 30 cycles of 30 s at 94°C, 30 s at 56°C, and 2 min at 72°C with a final elongation of 10 min at 72°C) was carried out in a 25 µl volume containing 3 µl of digested ligated products, 1X PCR Buffer II at pH 8.3, 1.5 mM of MgCl_2_, 80 µM of dNTP mix, 0.2 µM of each primer at 10 µM (EcoRI+1⁄MseI+1), and 0.5 U of AmpliTaq Gold DNA Polymerase (Applied Biosystems, Forster City, CA, USA). After performing a 1:20 dilution of the pre‐selective PCR products, a selective amplification was carried out in a 12.5 µl volume containing 2.5 µl of diluted pre‐selective PCR product, 1X PCR Buffer II, 2.5 mM of MgCl_2_, 80 µM of dNTP mix, 0.2 µM of each FAM‐labeled primer (EcoRI+3⁄MseI+3, EcoRI+3⁄MseI+3), 8 µg/ml BSA, and 0.5 U of AmpliTaq Gold DNA polymerase (10 min at 95°C; 13 cycles of 30 s at 94°C, 60 s at 65°C to 56°C, and 60 s at 72°C; 23 cycles of 30 s at 94°C, 60 s at 56°C, and 60 s at 72°C with a final elongation of 10 min at 72°C). These primer combinations were chosen based on preliminary tests according to Pompanon, Bonin, Bellemain, and Taberlet (2005), which resulted in clear banding patterns and sufficient variability. The PCR products of the EcoRI⁄MseI combinations were purified using columns of half 5% Sephadex G50 and half Sephacryl S200. The PCR products from each EcoRI⁄MseI primer pair were run separately for fragment length analysis; 1.5 µl of the FAM‐labeled products was mixed with 10 µl of HiDi formamide and 0.1 µl of Genescan ROX 500 size standard (Applied Biosystems) and then electrophoresed on an ABI PRISM 3130 capillary sequencer (Applied Biosystems).

AFLP profiles were analyzed using Peak Scanner software (Applied Biosystems); the profiles, for which the automated process failed to detect or attribute peak size, were processed manually. The binning and scoring of the profiles were performed using the *RawGeno* package (Arrigo, Tuszynski, Ehrich, Gerdes, & Alvarez, 2009) in R software (R_Development_Core_Team, 2011). We kept individuals within the 5%–95% quantile confidence interval around the peak number mean and kept the peaks that corresponded to fragments longer than 100 bp to limit size homoplasy (Arrigo et al., 2009; Pompanon et al., 2005). Bins that were not reproducible at least once within a group of replicates (four groups of replicates for each pair of primers and three to four replicates per group) were systematically removed. We calculated the “bin content information” criterion (Arrigo et al., 2009; Pompanon et al., 2005) for various filtering strategies and found that our conservative strategy retained the most information. A total of 93 individual plants were reliably scored for the presence or absence of 345 reliable and polymorphic loci, which were coded in a binary matrix.

The plant population genetic structure was inferred from AFLP data using STRUCTURE 2.3.2 (Pritchard, Stephens, & Donnelly, 2000), implementing an admixture model without a priori knowledge of the geographic provenance of individuals. The likelihood of the number of genotypic clusters (K) was estimated for K ranging from 1 to 20 with five runs for each K value with 50,000 burn‐in periods followed by 50,000‐step Markov chain Monte Carlo (Supporting Information Figure S1). Pairwise genetic distances were assessed with the Jaccard index.

### 2.4 Fungal ITS1 sequencing, clustering, and assignment

The ITS1 DNA region was amplified by PCR using DNA extracted from each soil core as previously described (Roy et al., 2013). Fungal DNA was amplified using the ITS5 (5′‐GGAAGTAAAAGTCGTAACAAGG‐3′) and the 5.8S_fungi primers (5′‐CAAGAGATCC′GTTGTTGAAAGTT‐3′) (Epp et al., 2012), extended in 5′ by sample‐specific tags of 8 nt in length to allow for parallel sequencing of multiple PCR samples. The PCRs (10 min at 95°C, 33 cycles of 30 s at 95°C, 15 s at 54°C, and 30 s at 72°C with a final step of 7 min at 72°C) were performed in a 25 µl volume containing 2.5 mM of MgCl_2_, 1 U of AmpliTaq GoldTM buffer, 20 g/L of bovine serum albumin, 0.1 mM of each dNTP, 0.26 µM of each primer, 2 U of AmpliTaq Gold DNA polymerase (Applied Biosystems, Courtaboeuf, France), and 10 ng of DNA template. The PCR products were purified, quantified (Qiaxcell), and pooled on an equimolar basis, and amplicons were sequenced in two sequencing runs on an Illumina MiSeq 2000 platform using 2x250‐bp paired‐end sequencing, yielding a total of 2,652,247 reads.

Bioinformatic processing of sequenced amplicons used the *OBITools* suite (Boyer et al., 2016). Reads were quality‐filtered, and ITS amplicons were reconstructed by merging the 3′ and 5′ pair‐end reads using *solexapairendnull*. We used *obigrep* to discard merged reads with an alignment score ≤20 (corresponding to a perfect match on ≤5 bases for reads with the highest Phred score) together with those containing ambiguous nucleotides, or errors in the primer sequence or the sample tag. The remaining merged reads were assigned to their sample of origin by their terminal tags using *ngsfilter*, which trims primers and tags away from the sequence, and identical reads were counted within each sample using *obiuniq*. Finally, singletons were removed following Coissac, Riaz, and Puillandre (2012), together with sequences shorter than 66 nt (the smallest one in our taxonomic database, see below), and an abundance table was built using *obitab*.

To cluster sequences into MOTUs, we first aligned them pairwise using a global Needleman–Wunsch algorithm implemented in the Sumatra package (https://metabarcoding.org/sumatra). Each pair yielded a similarity index equal to the longest common subsequence normalized to the alignment length. Indices were then used to build clusters sharing at least 98% similarity (Lentendu et al., 2011) using the nonhierarchical Markov clustering method (MCL; van Dongen, 2000; Zinger, Shahnavaz, Baptist, Geremia, & Choler, 2009).

The taxonomic assignment of MOTUs used a custom ITS1 database derived from the UNITE database (release V7, 2017–01) using *ecoPCR*, which extracts sequences flanked by two input primers. The most represented sequence of each MOTU was aligned against this ITS1 database using the fasta35 algorithm (Pearson, 2000) implemented in the *ecotag* program, and only environmental sequences identical at a minimum of 80% to a reference sequence were assigned. Possible lifestyles were deduced from the MOTU taxonomy using FUNGuild (Nguyen et al., 2016).

Nine samples with fewer than 900 reads were removed, and MOTUs with less than 10 reads or unassigned to the Fungi kingdom were discarded. The final dataset contained 175 samples and 1,311 fungal MOTUs across 1,016,938 reads. The sequences and metadata of the samples are available from EMBL under ENA entry PRJEB14389.

### 2.5 Statistical analyses

MOTU read counts per sample were transformed to relative abundance. To assess the compositional changes between soil fungal communities, we summarized pairwise Bray–Curtis dissimilarities by non‐metric multidimensional scaling (NMDS), using *vegan* (Oksanen et al., 2013). Environmental fitting was used to visualize the correlation between variation in soil parameters and variations in the fungal community. Dissimilarities between cognate bare and cushion soils were calculated by comparing each cushion soil to each bare soil within each sampling site.

To rank the predictors of community composition in cushion soils, we implemented a variance partitioning approach based on dissimilarity matrices in which fungal pairwise dissimilarities were measured using the Bray–Curtis index, either on fungal frequencies or on their square roots; the latter (Hellinger transformation) minimizes the impact of the most abundant MOTUs. Plant genetic dissimilarities were calculated using the Jaccard index on AFLP fingerprints. Additionally, Euclidian distances were used for soil abiotic environment (using reduced chemical data combining nitrogen, carbon, water, nitrate and ammonium contents, and pH values), both beneath and outside cushions and for geographic positions. Instead of using a traditional regression analysis of variance, we chose a generalized additive model (GAM) that deals with potential nonlinear relationships between pairwise beta‐diversities and environmental distances (Ferrier, Manion, Elith, & Richardson, 2007; see Supplemental data) and thus increases the predictive power. The pure and joint explained variance of each predictor (either plant dissimilarities or environmental distances) was calculated using Nagelkerke's *R*‐square: The pure fraction represents the effect of a variable after accounting for the effects of all other predictors. We also extended the general formula for two variables to the case of four variables and represented the pure and joint explained variance using a Venn diagram.

To test for significant differences between taxonomic abundances beneath and outside cushions, we fitted two linear mixed models using *lme4* (Bates, Maechler, Bolker, & Walker, 2015): a null model and a model including, as a fixed factor, the location beneath cushion or in bare soil. The two models included the sampling site as a random factor. The models were then compared in an ANOVA test, and *p‐*values were corrected for multiple testing using the false discovery rate (Benjamini & Hochberg, 1995). Fungal diversity was quantified using the inverted Simpson index.

To identify MOTUs that were recurrently associated with plants, we grouped samples into six habitats combining the granite/limestone bedrock type, the local plant genetic cluster (see Results), and the beneath/outside cushion location. For each MOTU, we counted occurrences over a 0.1% frequency threshold in each habitat and performed a chi‐square test of independence. The *p‐*values were obtained through 10,000 permutations of the original matrix and corrected for multiple testing. We kept those MOTUs that were significantly and positively associated with at least one cushion habitat (*p‐*value < 0.05; Pearson residual > 2) but with no bare soil habitat.

## 3 RESULTS

### 3.1 Genetic structure of *Silene acaulis* populations

Two genetic groups of plants were revealed by population structure inference (Supporting Information Figure S1) and the ordination of distances between AFLP fingerprints (Figure 2b). Most samples had at least 80% of their AFLP markers belonging to a single genotypic group (Figure 2a). This criterion allowed us to define two plant genetic clusters and left only four ambiguous plant individuals. The two genetic clusters matched with subspecies determination (Figure 2a), namely the dense cushion *exscapa* and the loose‐cushion *longiscapa* (Figure 2), and they were named accordingly.

**Figure 3:**
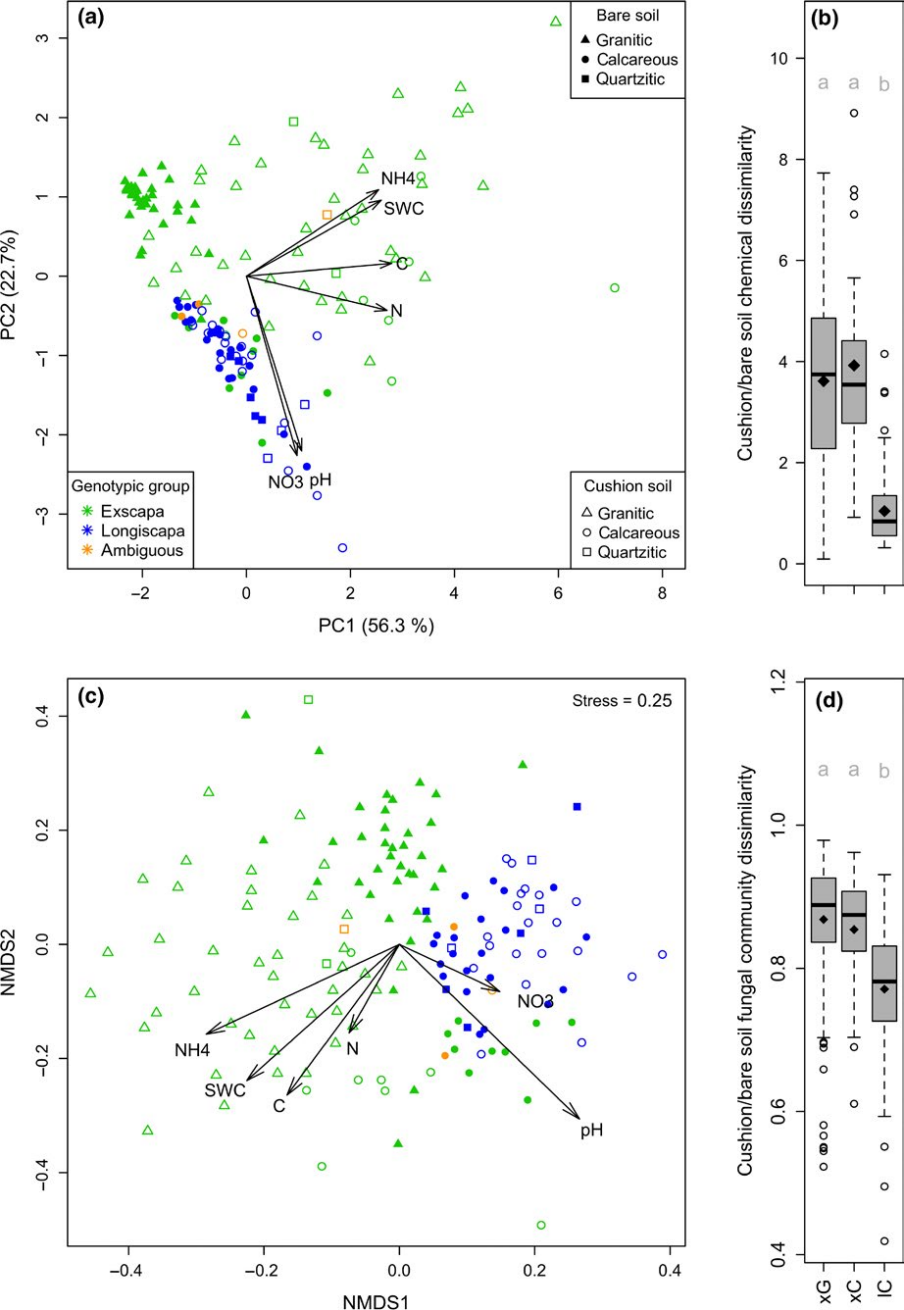
Abiotic and biotic soil engineering. (a) Soil abiotic parameters ordinated by PCA. Arrows represent the correlations of chemical variables to axes magnified three times. (b) Dissimilarities in soil abiotic parameters between cognate bare and cushion soils. (c) Fungal community dissimilarities ordinated by NMDS; vectors indicate correlations of environmental variables with NMDS axes (all significant in a permutation test, *p* < 0.001). (d) Fungal communities’ dissimilarities between cognate bare and cushion soils. Black diamonds indicate means, and distinct gray letters indicate means that differ significantly (Wilcoxon test, *p*<0.001)

At each of the 19 sampling sites, only one genetic cluster was found, except for the two sites containing ambiguous individuals (Figure 2a). The *longiscapa* genetic cluster only occurred on calcareous bedrock (limestone), except for one population found on quartzite (CIIIQ, Figure 2a) for which the abiotic environment was close to that of the calcareous environments (Supporting Information Table S1; Figure 3a). By contrast, the *exscapa* genetic cluster was found on both calcareous and siliceous (granite and quartzite) bedrocks. Although *exscapa *culminated at higher elevations than *longiscapa*, six populations of *exscapa* and three populations of *longiscapa* occurred within the same elevation range (2,500–2,700 m a.s.l.; Supporting Information Table S1, Figure 2a).

### 3.2 Differences in soil abiotic engineering by the two plant genetic clusters

The PCA ordination of soil chemical properties revealed two axes totalizing 79% of the total variability (Figure 3a). Axis 1 was related to nutrient and water contents, and Axis 2 was related to soil pH and nitrate content. Cushions from *exscapa* genetic cluster induced a strong shift of soil chemical properties along Axis 1 from bare to cushion soils. This shift was smaller for *longiscapa* genetic cluster (Figure 3a,b). Supporting Information The contents in nitrogen, carbon, water, and ammonium all increased on both granitic and calcareous bedrocks beneath *exscapa* plants, and soil became less acidic on granite (Supporting Information Figure S3). For soil beneath *longiscapa* cushions, the increase in nutrient contents was weaker, although significant for the carbon and ammonium contents (Supporting Information Figure S3).

### 3.3 Differences in the engineering of fungal communities by the two plant genetic clusters

**Figure 4:**
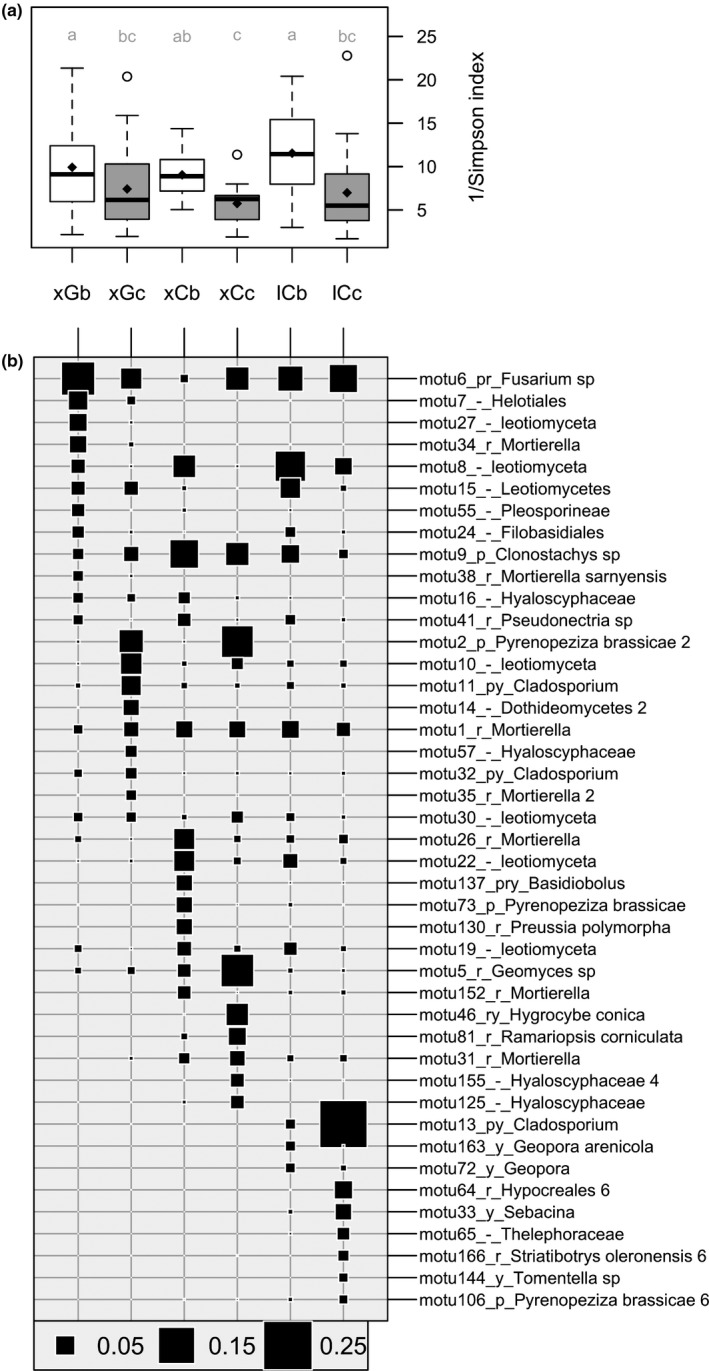
Fungal diversity and turnover across habitats. Samples were grouped into six habitats combining the plant genotype (“x” for *exscapa *and “l” for *longiscapa*), the bedrock type (“G” for granitic and “C” for calcareous), and the location (“b” for bare soil and “c” for cushion soil). From left to right, *n* = 40, 43, 12, 11, 22, and 20. (a) Simpson indices for fungal MOTU diversity. Diamonds indicate means, and gray labels sharing no letter indicate statistically different means (Wilcoxon test, *p* < 0.05). (b) Major MOTUs**. **In each habitat, the 12 most abundant MOTUs were selected (this represented at least 53% of the reads in all cases). The MOTU taxonomic assignment is preceded by the FUNGuild trophic mode: r for saprobes, p for pathotrophs, and y for symbiotrophs. The final numbers 2, 4, and 6 denote those MOTUs for which the number of occurrences was significantly higher in a plant habitat beneath *exscapa* on granite, *exscapa* on limestone, and *longiscapa *on limestone, respectively, than in other habitats (χ2 test, see Methods)

NMDS ordination of fungal community dissimilarities revealed a sharp difference in community composition on the two main geological substrates, which correlated with pH and nitrate contents (Figure 3c). On top of this bedrock‐related divergence, fungal communities of *exscapa* cushions from both mountain ranges segregated away from all other habitats, which correlated with increased soil contents in carbon, total nitrogen, ammonium, and water. For *longiscapa*, the shift in community composition differed from the one observed for *exscapa* on limestone (Supporting Information Figure S2). From bare to cushion soils, fungal dissimilarities were strong for both subspecies (Figure 3d). The shifts in community composition included a consistent decrease in fungal diversity beneath the cushions compared to bare soils (Figure 4a). This diversity reduction came along with a strong MOTU turnover, which was modulated differently for the two genetic clusters and the two bedrock types (Figure 4b).

### 3.4 Links between plant genetic distances and fungal beta diversity

**Figure 5:**
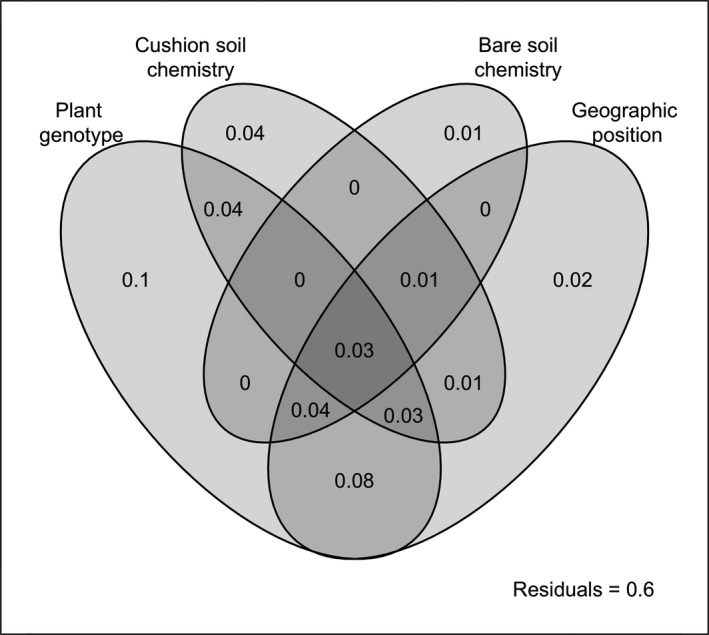
Predictors of variation of the fungal communities hosted by cushions. Pairwise dissimilarities in a set of 64 cushions were used to partition the variance of fungal communities using GAM (see Methods). The pure and joint effects of the different explanatory variables on fungal variation are indicated

The variations in the composition of fungal communities among cushion soils were best explained by the combined variations in plant genotype, soil chemical composition beneath and outside the cushions, and geographic position (Figure 5), which accounted together for 40% of the total variability. This variability was 2% more than when the geographic position was dismissed at the profit of elevation (Supporting Information Figure S4). The largest pure effect (10%) was that of the genetic distances between plants (Figure 5), and large plant genetic distances reflected chiefly differences between individuals of different genetic clusters (Figure 2b). The largest shared part (8%) was explained together by plant genetic and geographic distances (Figure 5), reflecting the geographic separation of the two plant clusters. When using a metric minimizing the impact of the most abundant MOTUs, the pure effects of geographic and genetic distances were equal (Supporting Information Figure S4). This discrepancy suggests that each plant cluster selectively recruited a few MOTUs that rarely occur in bare soils but became highly abundant beneath cushions.

### 3.5 Taxonomic identification of fungal sequences associated with the cushion habitats

**Figure 6:**
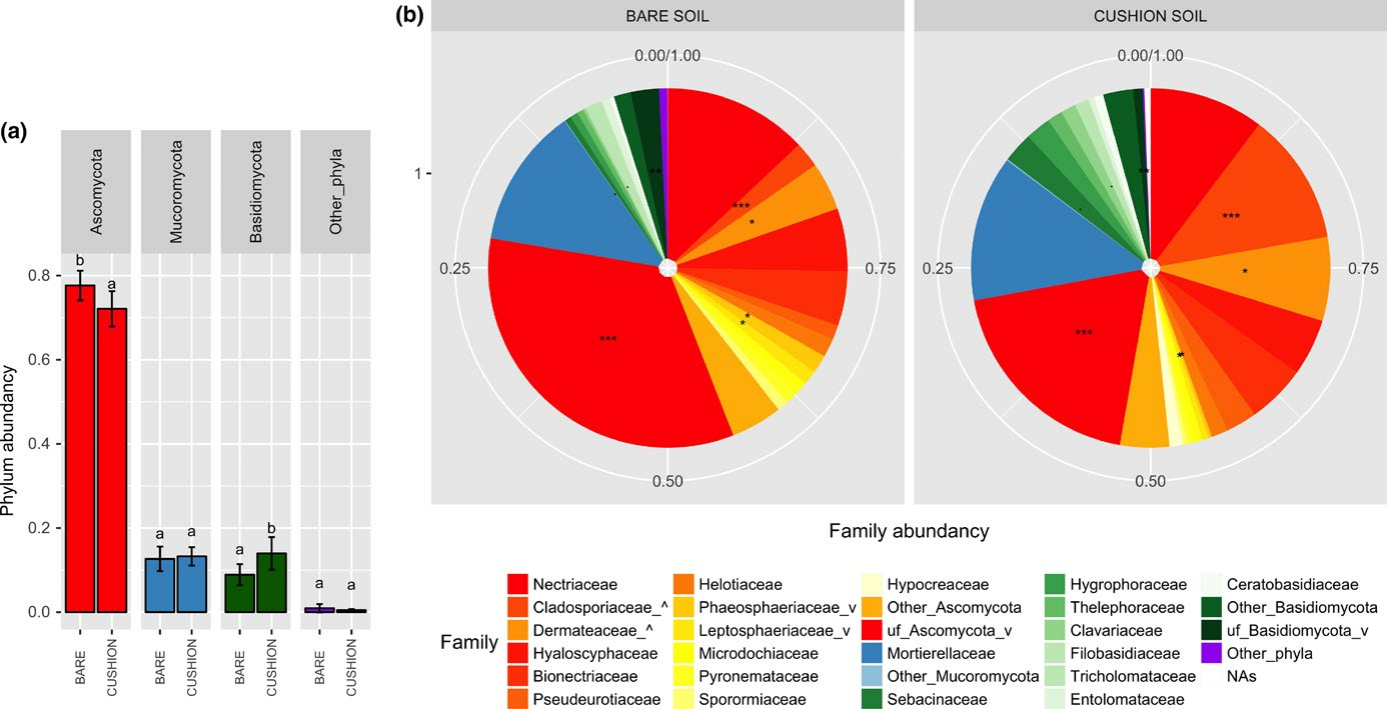
Composition of the fungal communities. Bare soils (*n* = 86) and cushion soils (*n* = 90) were compared. (a) Phylum abundances: Error bars show SE; letters differ when means are significantly different (*p* < 0.05). (b) Fungal family abundances. Ascomycota are in the red‐to‐yellow color range, Mucoromycota appear in blue, and Basidiomycota are in the green range. Families representing <1% of the total reads were pooled. MOTUs without assignment at the family level were pooled by phylum (“uf” prefix); “NAs” indicate MOTUs of unknown phylum. Families whose frequencies vary from bare soils to cushion soils bear a suffix (“_^”, increase; “_v”, decrease); the significance of the difference is indicated on sectors (“.”; “*”; “**”; “***” for *p* < 0.1, 0.05, 0.01, and 0.001, respectively)

Ascomycota largely dominated all fungal communities but decreased beneath cushions vs. bare soils, while Basidiomycota significantly increased (Figure 6a). The latter included a trend for Sebacinaceae to increase (Figure 6b; 2.7% beneath vs. 0.6% outside). In both phyla, the fraction of fungi unassigned at the family level was high in bare soils and decreased strongly in cushion soils (Figure 6b). Remarkably, the abundance of Cladosporiaceae and Dermateaceae (Ascomycota) increased largely and significantly beneath the cushions, cumulating in 19.4% of the reads in cushion soils vs. 6.6% in bare soils. The nearly doubled frequency of Dermateaceae was entirely caused by MOTUs assigned to *Pyrenopeziza brassicae *(Supporting Information Table S2).

We observed a strong turnover of fungal MOTUs across the six habitats (*exscapa* cushions on granite and limestone, *longiscapa* cushions on limestone, and their bare soil cognates). Among 43 MOTUs abundant in at least one habitat (Figure 4b), only six (MOTU‐1, MOTU‐5, MOTU‐6, MOTU‐8, MOTU‐9, and MOTU‐11) reached at least 0.2% of the reads in all six habitats. Remarkably, however, none of the major 43 MOTUs increased in abundance or occurrence number beneath each of the three cushion habitats with concerning the cognate bare soils, and this holds true even when considering limestone alone. Instead of cushion generalists, we noted strong differences between the two plant clusters. For instance, MOTU‐2 (*Pyrenopeziza brassicae*), MOTU‐10 (Leotiomycota), MOTU‐109 (*Mortierella*), and MOTU‐141 (Dothideomycetes) increased in abundance or occurrence beneath *exscapa* cushions on both granitic and calcareous bedrock types (Figure 4b, Supporting Information Figure S5). Conversely, beneath *longiscapa* cushions, the *Cladosporium* MOTU‐13 accounted for 24% of the sequences but was below 0.03% beneath *exscapa* cushions regardless of the bedrock type. Two other *Cladosporium,* MOTU‐11 and MOTU‐32, increased only beneath *exscapa* cushions on granitic bedrock (Figure 4b). MOTU‐2 and MOTU‐106, both assigned to *P. brassicae*, showed opposite cluster associations.

The contrasted fungal communities included both putative biotrophic and saprotrophic fungi. MOTU‐2, MOTU‐11, MOTU‐13, MOTU‐21, and MOTU‐106 likely belong to phytopathogen fungal species, whereas MOTU‐33 (*Sebacina*), that increased beneath *longiscapa *cushions (Figure 4b), is likely symbiotic. Among putative saprobes, MOTU‐35, MOTU‐83, MOTU‐109 (*Mortierella*), MOTU‐5 (*Geomyces*), MOTU‐81 (*Ramariopsis*), and MOTU‐101 (*Hymenoscyphus*) were associated with *exscapa* cluster, whereas MOTU‐64 (*Hypocreales*) and MOTU‐166 (*Striabotrys*) were associated with *longiscapa* cluster (Figure 4b; Supporting Information Figure S5).

## 4 DISCUSSION

We have studied the fungal communities of a foundation cushion plant in a microevolutionary context across contrasting environmental conditions, such as different bedrocks. Genotyping of *S. acaulis *cushions delineated two genetic clusters, one of which can be found both on siliceous and limestone bedrock, while the other one is restricted to limestone. This unique situation, despite asymmetries in the dataset, allows highlighting the key influence of plant genotypes on soil engineering, both in abiotic modifications and in the filtering of fungal species.

### 4.1 The two plant subspecies differentially engineer their soil environment

As previously reported (Roy et al., 2013), cushions engineer a soil enriched in carbon and ammonium, two key soil nutrients that are mainly derived from plant litter; here, we found that this edaphic improvement is clearly stronger for the *exscapa* cluster. These differences in abiotic soil engineering are likely to derive from differences in cushion architecture. A causal link between the nurse plant stature and its engineering effects has been demonstrated in a dominant coastal shrub (Crutsinger et al., 2014). The *exscapa* subspecies displays a denser architecture than the *longiscapa* one, limiting litter dispersion and certainly providing a higher capacity to limit water loss. Differences in cushion architecture between subspecies most likely have a heritable basis because they correspond to distinct genotypic groups and remain observable over a broad elevation range and on distinct bedrocks. Our sampling, however, does not encompass the whole ecological range of each subspecies and therefore does not explore their entire vegetative trait plasticity (Bonanomi et al., 2016), making it difficult to tease apart the respective influences of phenotypic plasticity and genetic differentiation.

Fungal communities strongly varied from bare soil to cushion soils, pointing to the selective filter exerted by cushions on fungi. This filtering is highlighted first by a decrease in fungal α‐diversity and by strong shifts in fungal composition. These changes were again stronger for the *exscapa *cluster and seem to be related to the plant subspecies rather than to the environment because they occurred with the same amplitude on the two contrasted bedrocks where *exscapa* plants were found. *S. acaulis* cushions improve edaphic properties but can also buffer soil moisture and alter the temperature regimes (Bonanomi et al., 2016; Molenda et al., 2012). This new microenvironment may indirectly favor the growth of new, especially saprophytic, fungal species. There is also a clear influence of the bedrock, as illustrated by the *exscapa* cushions, which remained largely different in fungal composition on granite or on limestone. Bedrock, as seen by bare soil chemistry, contributed little as a pure part to the variability of fungal composition but much more to the fractions shared with a geographic position, owing to the spatial separation of limestone and granite sites.

### 4.2 Indirect and direct mechanisms of fungal recruitment by cushions

For the three cushion habitats (*exscapa* on granite and limestone, and *longiscapa* on limestone), comparison to bare soils revealed both a strong turnover for putatively saprophytic and biotrophic fungi. In both classes, MOTUs were nearly always recruited by one plant subspecies only, including on the common limestone bedrock. The contribution of cushion soil nutrients to fungal variations owes much to plant engineering, thus highlighting an indirect effect of plant genetics most likely to impact saprobes. Saprobes recurrently associated with a single host could respond to different degrees of soil engineering from the plant litter, be it in the content in organic matter, nutrient (Hanson, Allison, Bradford, Wallenstein, & Treseder, 2008; Treseder & Lennonb, 2015), or water (Crowther et al., 2014). For instance, *Geomyces* (Ascomycota) possibly bloomed beneath *exscapa* cushions because they occupy the highest elevation on limestone since this genus includes psychrophilic species able to profit from a variety of organic substrates (Hayes, 2012). Saprobes could also be host‐specific epi‐ or endophytes turning into decomposers upon plant leaf senescence (Osono, 2002; Voriskova & Baldrian, 2013; Zhou & Hyde, 2001).

Plant genetic distance was the best predictor for fungal variations for major MOTUs; therefore, plant genetic identity directly explained a part of the variance in community composition that was not explained by ecosystem engineering. For less abundant MOTUs, the geographic distance was, however, as strong a predictor as plant genetic distance, possibly reflecting stochastic recruitment processes (Dumbrell, Nelson, Helgason, Dytham, & Fitter Alastair, 2009). The plant genetics direct effect likely exerts through the symbiotic and pathogenic fungal communities. Among symbionts, we observed *Sebacinae* (Basidiomycota), a family of root endophytic and ectomycorrhizal fungi (Weiß, Waller, Zuccaro, & Selosse, 2016). The former type of association is more likely since *S. acaulis* is poorly mycorrhized (Cripps & Eddington, 2005; Schmidt, Sobieniak‐Wiseman, Kageyama, Halloy, & Schadt, 2008).

Among putative pathogenic fungi, we observed opposite plant subspecies specificities within MOTUs belonging to the same genus, *Cladosporium* (Bensch, Braun, Groenewald, & Crous, 2012), or to the same species (*P. brassicae, *Dermateceae; Rawlinson, Sutton, & Muthyalu, 1978). MOTUs assigned to *Cladosporium* and Dermateaceae have been detected within the stems and leaves of *S. acaulis* in the Arctic (Zhang & Yao, 2015). This strongly suggests that direct interactions between the fungus and living plant tissues account for their recruitment in alpine cushion soils and is consistent with the strong co‐structure between phyllosphere fungal composition and intraspecific population genetic structure in beeches (Cordier, Robin, Capdevielle, Desprez‐Loustau, & Vacher, 2012) as well as with the phylogenetic signal in the association of tropical trees with fungal pathogens (Gilbert & Webb, 2007). These narrow host ranges might result from a local co‐evolutionary arm race (Chappell & Rausher, 2016) or from historical contingencies if the two plant subspecies have distinctive biogeographic origins and each imported their cohort of biotrophic fungi, as described for *Carex curvula* (Geremia et al., 2016).

The high abundance of putative pathogens beneath seemingly healthy cushion plants points to a reduced pathogenic virulence that may result from long‐term associations. Pathogen virulence is predicted to decrease with increasing host lifespan (van Molken & Stuefer, 2008), and plant–fungi interactions may shift from negative to positive with elevation (Defossez et al., 2011). The cost for the plant to host endophytic fungi has been shown to be weak (Kia et al., 2016). Fungi associated with cushion plants may, for instance, offset their fitness costs because they reduce herbivore or virulent phytopathogen attacks or increase plant tolerance to abiotic stress (Rodriguez, Redman, & Henson, 2004). In a situation where nutrients are scarce, there also might be an evolutionary trade‐off between the cost of hosting a biotrophic fungus and the accelerated nutrient cycling by the same fungus in the litter. Such a trade‐off has been predicted for insect grazers (de Mazancourt & Loreau, 2000).

### 4.3 Ecological implications

Our results suggest that the plant evolutionary dynamic of cushion plants strongly impacts the fungal composition of alpine ecosystems, possibly through diverging morphological traits. This extended phenotype sensu Whitham (Whitham et al., 2003) may have implications for plant succession. Cushion plants, including *S. acaulis,* help secondary plant species to settle in the landscape (Badano et al., 2006; Bonanomi et al., 2016). This settling may occur through reduction in physical stress (Bertness & Callaway, 1994), but the presence of antagonist fungi may also be important in this process. The Janzen‐Connell hypothesis (see Bever et al., 2012 for a review) proposes that microorganisms specific to a plant species allow plant coexistence by reducing the fitness of their host and are, thus, at the origin of biodiversity in plant communities. In addition, it was shown that symbiotic root endophytes of cushion plants increase the fitness of the facilitated plants (Molina‐Montenegro et al., 2015).

Plant–fungi interactions remain an overlooked component in the natural history and the present functioning of cold‐adapted ecosystems as well as their responses to environmental changes. Sampling along the entire ecological range of *S. acaulis* subspecies and other cushion plant species, together with the determination of fungi living in soil vs. plant tissues, may reveal the contributions of plant evolutionary dynamics and functional traits on fungal engineering.

## AUTHOR CONTRIBUTIONS

S.L., S.I., C.A., P.S., R.A.G., J.R., and J.C.C. planned and designed the research. J.R. performed the PCR. M.B. and D.R. performed the plant AFLP. S.L., C.A., P.S., S.I., and J.R. conducted the fieldwork. J.R., J.M.B., R.A.G., M.G., and M.O. analyzed the data, and J.R., R.A.G., S.L., P.C., J.C.C., and S.I. interpreted the results. J.R., R.A.G., and J.M.B. wrote the manuscript with input from all authors. We thank the CNRS‐LECA for providing funding to a group of young researchers to run the “Vertical Ecology” project.

## DATA ACCESSIBILITY STATEMENT

Data and R‐source code available from the Dryad Digital Repository: https://doi.org/10.5061/dryad.2v1m1fj.
