# Supplementary figures and images for: Differences in the fungal communities nursed by two genetic groups of the alpine cushion plant, Silene acaulis
Source: Ecol Evol. 2018 Nov 21;8(23):11568–81. doi: 10.1002/ece3.4606 (PMC6303776; doi:10.1002/ece3.4606)

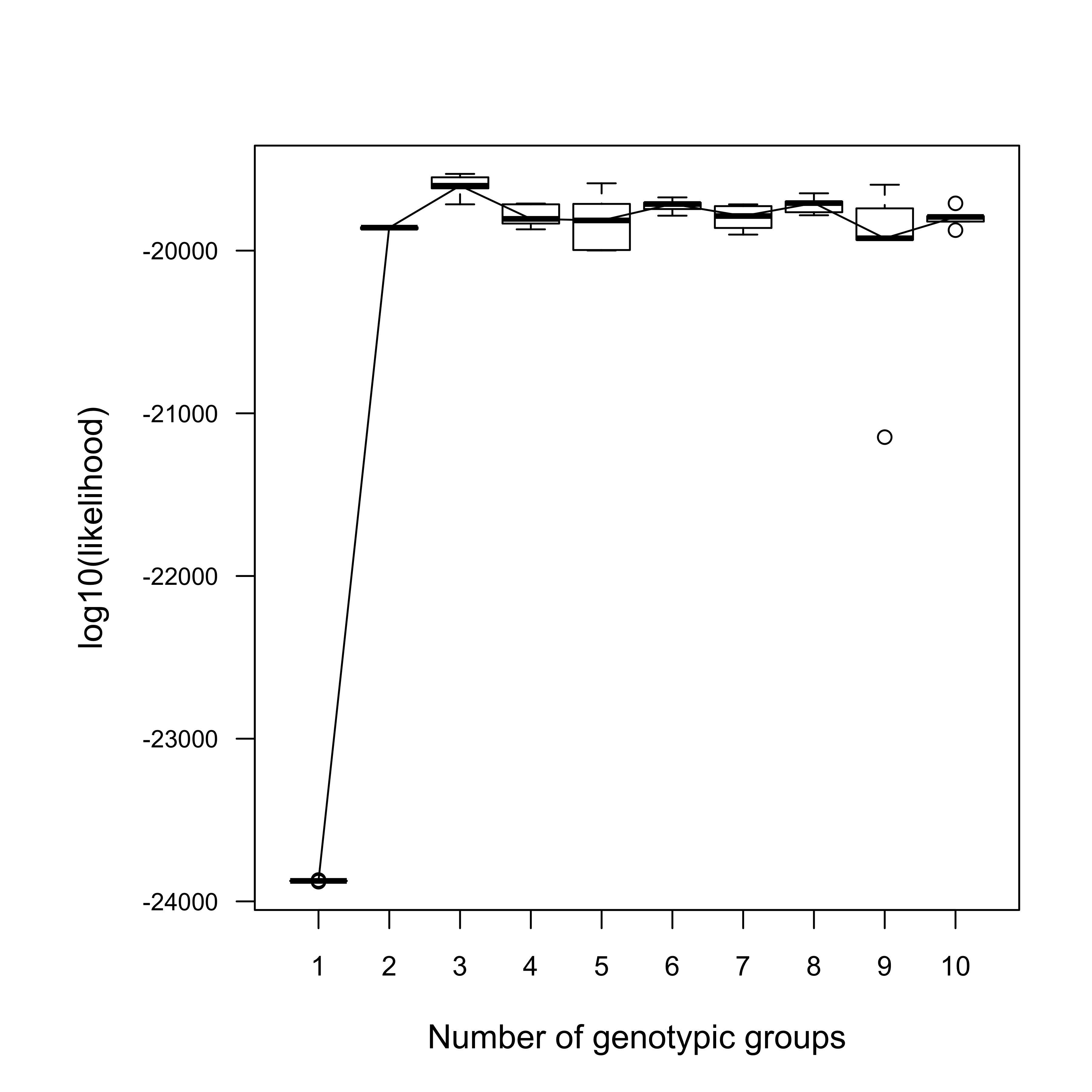

Supplement: Supplementary file 1 [file ECE3-8-11568-s001.tiff]

Bray\_Curtis fungal dissimilarities, 3D-NMDS

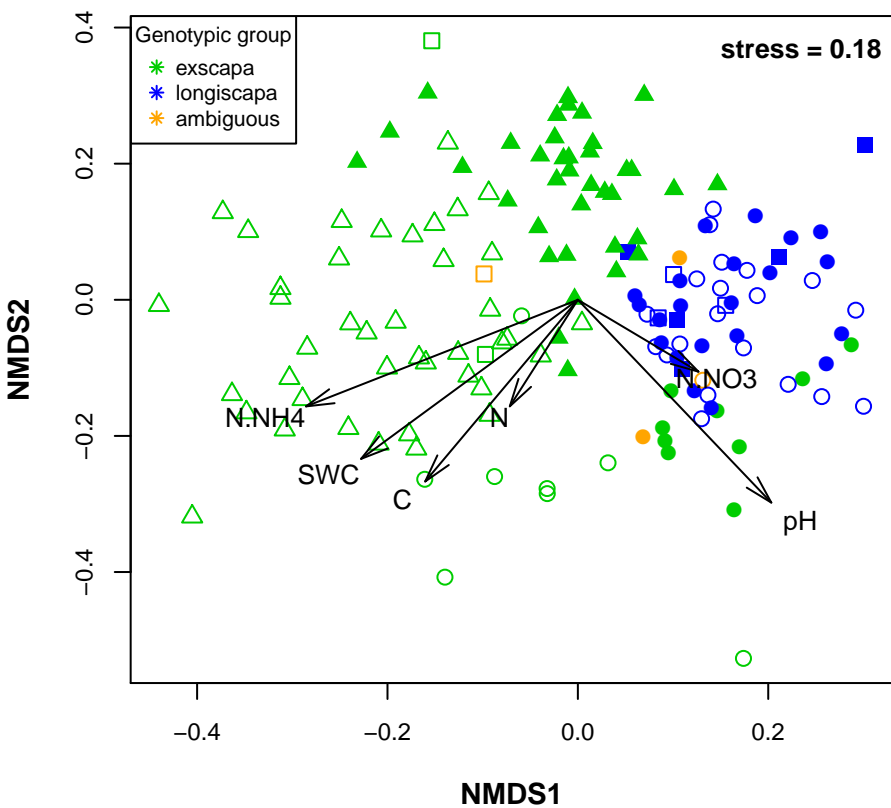

Bray\_Curtis fungal dissimilarities, 2D-NMDS

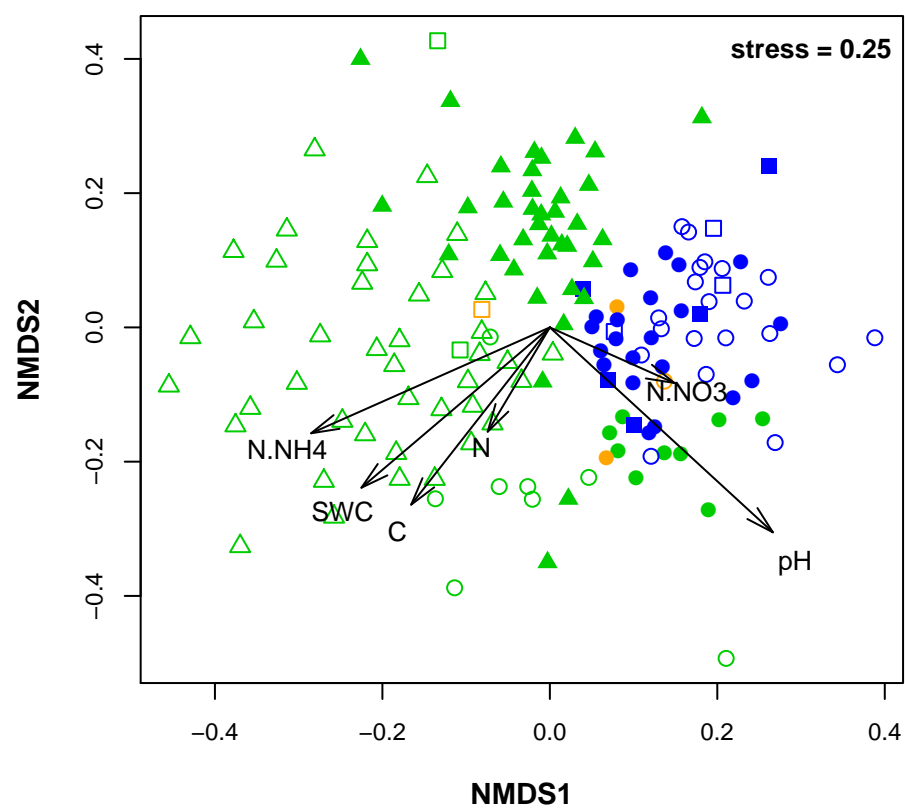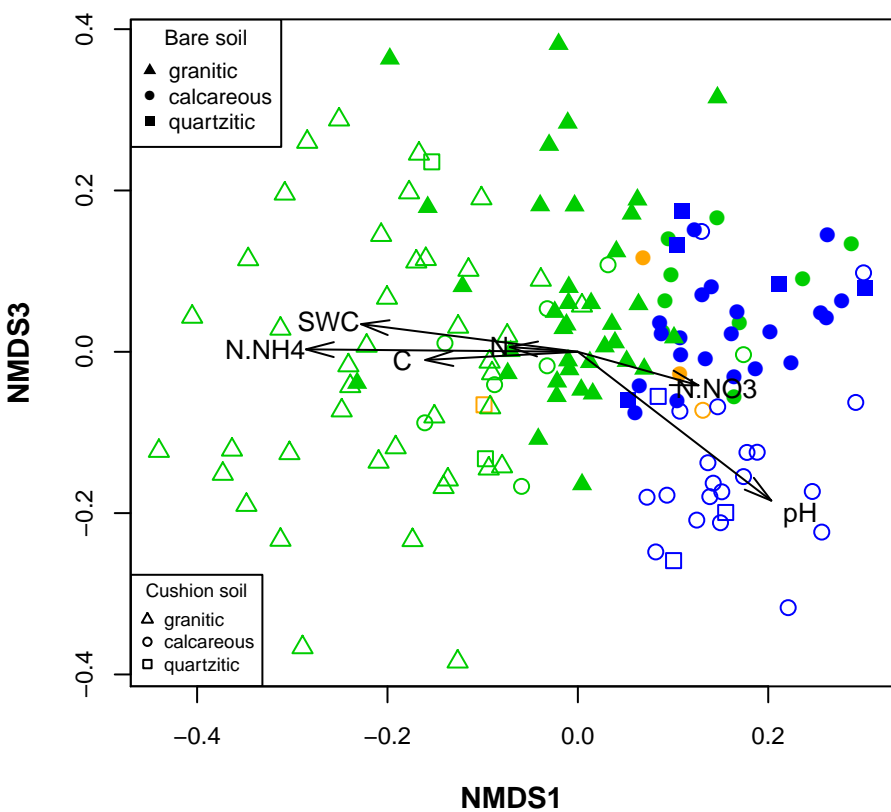

Supplement: Supplementary file 2 [file ECE3-8-11568-s002.pdf]

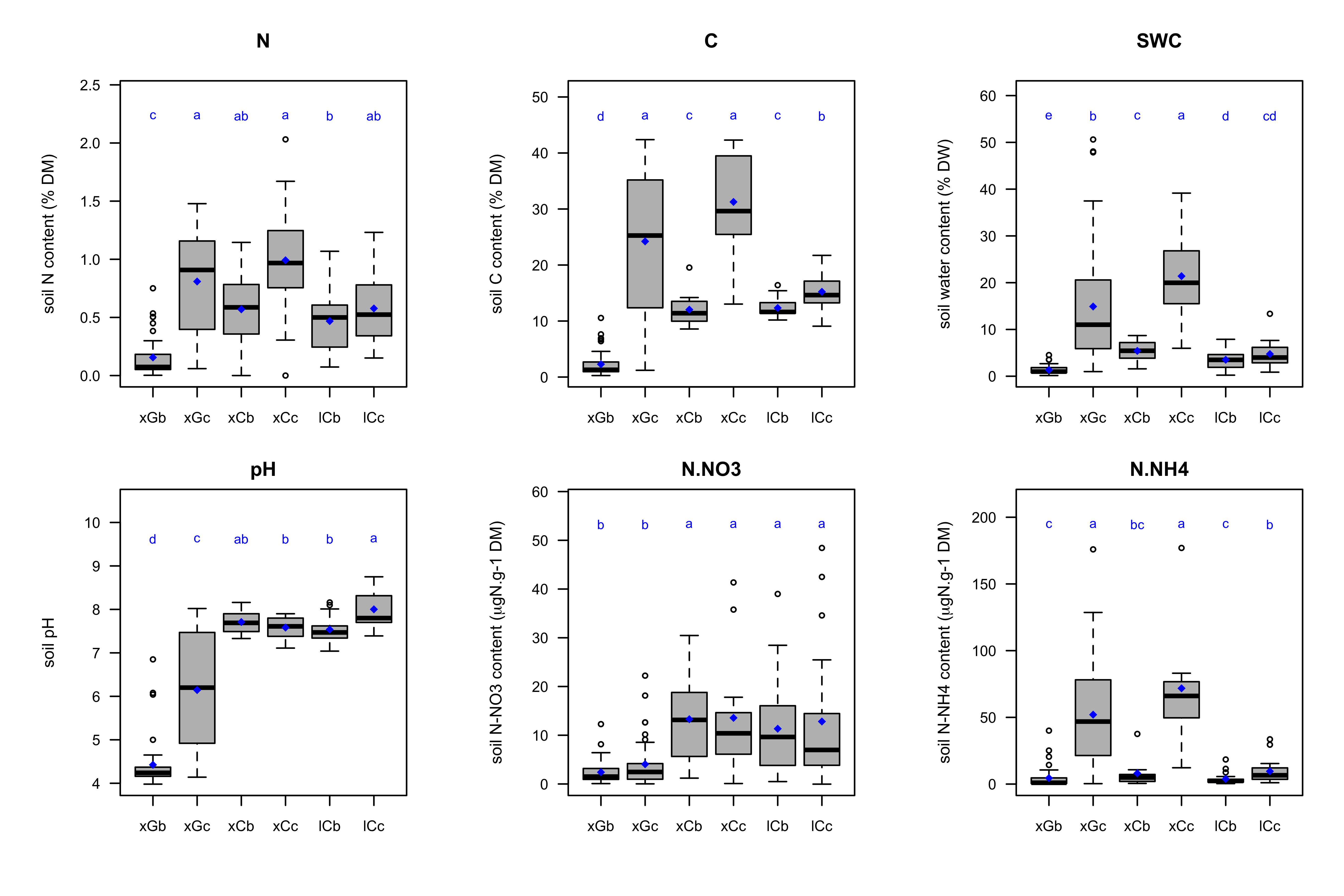

Supplement: Supplementary file 3 [file ECE3-8-11568-s003.tiff]

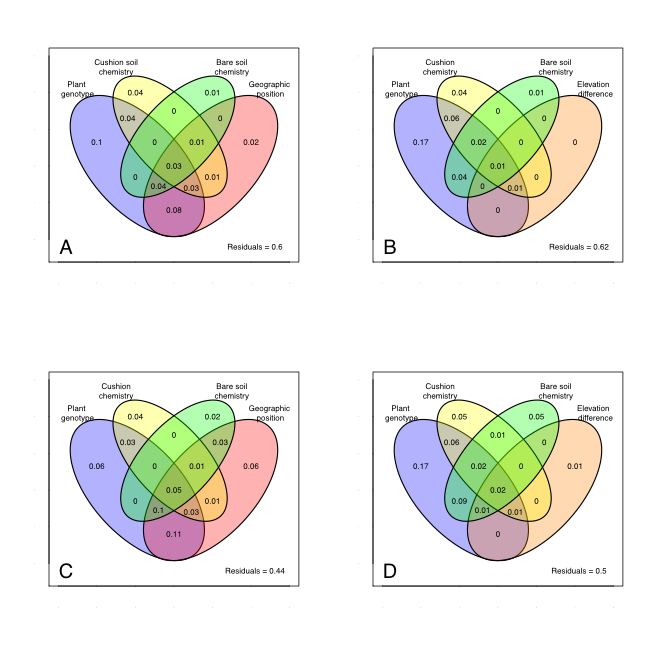

Supplement: Supplementary file 4 [file ECE3-8-11568-s004.tiff]

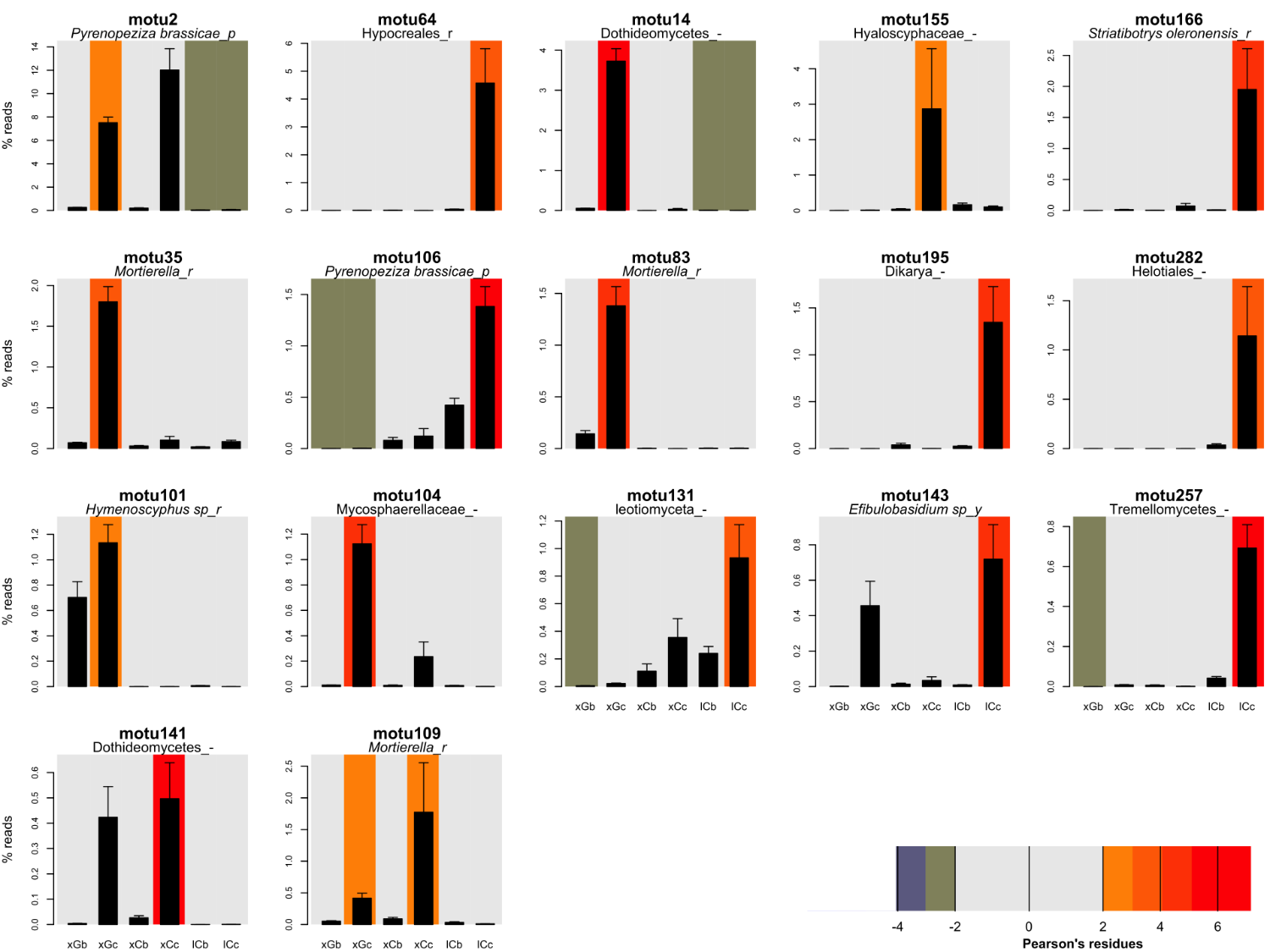

Supplement: Supplementary file 5 [file ECE3-8-11568-s005.tiff]
