# Supplementary material for: Differences in the fungal communities nursed by two genetic groups of the alpine cushion plant, Silene acaulis
Source: Ecol Evol. 2018 Nov 21;8(23):11568–81. doi: 10.1002/ece3.4606 (PMC6303776; doi:10.1002/ece3.4606)
Supplement: Supplementary file 8 [file ECE3-8-11568-s008.docx]

**LEGEND TO SUPPLEMENTAL FIGURES**

**Figure S1. Choice of the number of plant genotypic groups.** Likelihood of the models splitting the *S. acaulis* AFLP dataset into K genotypic groups as computed in STRUCTURE.

**Figure S2. Fungal community dissimilarities ordinated by NMDS in a 3D- (left) or 2D space (right).** Vectors indicate correlations of environmental variables with NMDS axes (all significant in a permutation test, P<0.001). The right panel reproduces Figure 2D to help comparison. Note lower NMDS stress in the 3-dimension ordination.

**Figure S3. Soil abiotic characteristics across habitats.** Samples were grouped into six habitats combining the plant genotype (‘x’ for *exscapa*, ‘l’ for *longiscapa*), the bedrock type (‘G for granitic, ‘C’ for calcareous), and the location (‘b’ for bare soil, ‘c’ for cushion soil). Populations on quartzite were removed. Mean values are marked by diamonds and were compared by Wilcoxon rank sum tests; they differ significantly when the blue labels share no letter (p<0.05). From left to right, *n*= 40, 43, 12, 11, 22, 20.

**Figure S4. Partition of variation of the fungal communities hosted by cushions.** Pairwise dissimilarities were computed using 64 samples and fitted using generalized additive models (GAM, see Methods). Fungal dissimilarities were computed before (panels **A, B**) or after Hellinger transformation of the MOTU frequencies (panels **C, D**; Hellinger transformation converts frequencies into their square roots). The pure and combined fractions of the total fungal variance accounted for by the various predictors are indicated. Panel A reproduces Figure 5 to help comparisons.

**Figure S5. Fungal MOTUs recruited in cushion habitats.** MOTUs were selected in a χ^2^ test comparing their numbers of occurrences in the six habitats combining plant genotype (‘x’ for exscapa, ‘l’ for longiscapa), bedrock (‘G for granitic, ‘C’ for calcareous), and location (‘b’ for bare soil, ‘c’ for cushion soil). Samples on quartzite were excluded; from left to right, *n*= 40, 43, 12, 11, 22, 20. MOTUs significantly and positively associated with at least one cushion- and with no bare soil habitat were retained. The χ2 Pearson residuals are indicated by color stripes (see palette), and the black histograms represent MOTU abundances across habitats (mean ± confidence interval 95%). The suggested trophic mode is indicated as suffix: ‘**r’** for saprobes, ‘**p’** for pathotrophs and ‘**y’** for symbiotrophs (‘**-**‘, unknown). MOTUs are sorted by decreasing mean abundance in their favored habitat, and only MOTUs with a frequence above > 0.5 in at least one habitat are displayed.
